# Hormone–receptor expression status of epithelial ovarian cancer in Ibadan, South-western Nigeria

**DOI:** 10.11604/pamj.2017.27.259.11883

**Published:** 2017-08-08

**Authors:** Mustapha Akanji Ajani, Ayodeji Salami, Olutosin Alaba Awolude, Abideen Olayiwola Oluwasola

**Affiliations:** 1 Department of Histopathology, Babcock University, Ilishan-Remo, Ogun state, Nigeria; 2 Department of Pathology, University College Hospital, Ibadan, Oyo state, Nigeria; 3 Department of Obstetrics and Gynaecology, University College Hospital, Ibadan, Oyo state, Nigeria

**Keywords:** Epithelial ovarian cancer, estrogen receptor, progesterone receptor

## Abstract

**Introduction:**

Epidemiological evidence strongly suggests that steroid hormones are implicated in the pathogenesis of ovarian cancer. Estrogen receptor (ER) and Progesterone receptor (PR) are prognostic indicators for a number of epithelial tumors and may play the same role in ovarian cancers. This study aims to evaluate the expression of ER and PR in epithelial ovarian cancer (EOC) in an African population and compare it with other prognostic factors such as age, International Federation of Gynaecology and Obstetrics (FIGO) stage, grade and histological subtype.

**Methods:**

Ninety cases of histologically confirmed EOC were reviewed. Immunohistochemistry was used to assess their ER and PR expression status and was then compared with other demographic variables using statistical methods, with level of significance set at p < 0.05.

**Results:**

30.2% and 8.3% of serous and mucinous carcinomas respectively were ER positive while 41.2% and 22.5% of both tumour types were PR positive. One of the two endometrioid carcinomas showed PR expression but neither were positive for ER. The only case of Brenner tumour in the series was ER positive but negative for PR. There was a significant association between ER and the histological subtypes (p = 0.042) while no significant association was found between PR expression and histological subtypes (p = 0.650). No significant association was found between hormone receptor status, age and stage of the EOC.

**Conclusion:**

The study showed a lower ER expression in serous carcinoma compared to large cohorts from developed countries. Future translational studies could be used to determine response of EOC to endocrine therapy.

## Introduction

Ovarian cancer causes more than 140,000 deaths worldwide every year and is the most lethal gynaecological malignancy in developed countries [1]. In the USA, this neoplasm ranks second among gynaecological cancers, yet it is by far the most lethal one, accounting for more than 15,000 deaths annually [2, 3]. Ovarian cancer is often known as “the silent killer,” its early detection is difficult because the ovaries are deep within the pelvis and initial symptoms are often ambiguous. The cancer goes undiagnosed until after the disease is far advanced and has spread throughout the abdomen or to distant organs. Nearly 75% of cases are diagnosed at an advanced stage, despite great efforts to develop reliable screening and prevention strategies [4, 5]. Majority of patients with ovarian carcinoma eventually succumb to disease due to development of treatment resistance. The introduction of cisplatin-based chemotherapy was initially thought to show improvement in the survival of patients with ovarian cancer, the percentage of recurrent disease is still high even in those patients who achieve a complete response to chemotherapy, so that more than 80% of patients with advanced stage of disease in Africa die within 5 years [2, 6, 7]. More effective treatment regimens and better early detection tools must be developed to improve morbidity and reduce mortality in ovarian cancer patients [3, 7, 8]. This has led to the development of “targeted” oncologic therapies that might ultimately be more effective and less toxic. Steroid hormone receptors expression in epithelial ovarian cancers has been proposed to have therapeutic and prognostic relevance, as is the case in breast cancer [7]. At present, the prognostic characterisation of ovarian cancer patients, based on clinicopathological parameters such as stage, histology, grade and residual tumour after surgery, seems to be inadequate, since patients with similar clinicopathological characteristics often experience different clinical outcome. Therefore, the identification of biological factors related to tumour aggressiveness could be relevant in order to identify patients with different prognosis and chance to respond to chemotherapy, thus allowing the selection, at the time of initial diagnosis, of high risk patients needing more aggressive therapy or alternative treatment and a closer follow-up [3, 8].

Among the biological parameters proposed as possible prognostic factors in ovarian cancer, much attention has been focused on endocrine factors and especially on steroid hormones and their receptors (estrogen and progesterone receptors). Epithelial tumours are most frequently associated with nulliparity, early menarche, late age at menopause and a long estimated number of years of ovulation [9, 10]. Different theories have been proposed as causal relationship between ovarian stimulation and neoplasia including Fathalla's incessant ovulation hypothesis which was initially thought to explain the pathogenesis of the tumour [11]. Recently however studies have shown that ovarian carcinomas arise via two main separate pathways, the high grade serous tumours are now known to arise from the fallopian tube fimbrial precursor lesions implanted on the ovarian surface [12–14]. Low grade tumours such as endometrioid carcinomas are believed to arise from areas of endometriosis [12–14]. Steroid hormones, primarily oestrogen and progesterone have been implicated in ovarian carcinogenesis. Oestrogens are major regulators of growth and differentiation in normal ovaries. Loss of heterozygosity at the 11q23.3-24.3 region which contains the PR gene has been associated with an elevated risk for ovarian cancer and poorer prognosis. Because high expression of ER and PR has been reported in EOC samples, it is hypothesized that expression patterns of ER and PR may be related to tumour behaviour, prognosis, or both [7, 15, 16]. Oestrogen may contribute to initiation and/or promotion of ovarian carcinogenesis. It is thus logical to speculate that the over-expression of ER should be associated with a poor prognosis. On the other hand, progesterone may offer protection against ovarian carcinoma development [17]. The ER and PR mediate the effects of female steroid hormones on proliferation and apoptosis of ovarian cancer cells [18]. Sieh et al have shown higher expression of ER in all ovarian carcinomas compared to PR expression. According to their study PR stained highest in endometrioid and low grade serous carcinoma but lowest for mucinous and clear cell carcinomas. Low grade serous carcinomas showed highest expression of ER followed by High grade serous carcinoma while clear cell carcinomas were least positive [18]. There has been no work done on the expression of ER and PR in ovarian cancers seen in females in Nigeria. This study aims to contribute to the body of knowledge by evaluating the expression of ovarian cancers in females seen in our environment.

## Methods

A total of 115 cases of histologically diagnosed epithelial ovarian cancers were seen in the hospital during the study period from January 2006 to December 2012. Out of these, ninety histologically confirmed cases were used in the study while the remaining twenty five cases which were metastatic cancers in the ovary and primary EOC in which slides were unsuitable or tissue blocks were unavailable were excluded from the study. Non-epithelial primary ovarian cancers were also excluded from the study. The tissues were obtained from archived surgical specimens of the department of Pathology at University College Hospital, Ibadan, obtained over a five year period. The demographic data and clinical history of the patients were obtained from the case notes, surgical daybooks, surgical pathology request forms, post-mortem records and Cancer Registry data. All the specimens were reviewed and their histological type, histological grade and FIGO staging was assessed. The histological type was determined on tissue sections according to 2014 World Health Organization (WHO) criteria [19]. The microscopic grading of Shimizu and Silverberg, which assesses architectural pattern, nuclear pleomorphism and mitotic activity, was utilized to grade and assign the tumours into different histological grades. Predominant architectural pattern was graded as follows: Glandular = 1, Papillary = 2, and Solid = 3. Nuclear pleomorphism was graded as Slight = 1, Moderate = 2, Marked = 3 and Mitotic activity was graded by counting mitotic figures per 10 high- power fields (in the most active regions): 0-9 = 1, 10-24 = 2, and ≥ 25 = 3. The total scores were summed and the final grades were as follows Grade 1 (well differentiated) = total score 3-5, Grade 2 (moderately differentiated) = 6 or 7, Grade 3 (poorly differentiated) = 8 or 9 [20]. FIGO Classification was used to determine the stage of the tumour.

**Figure 1:**
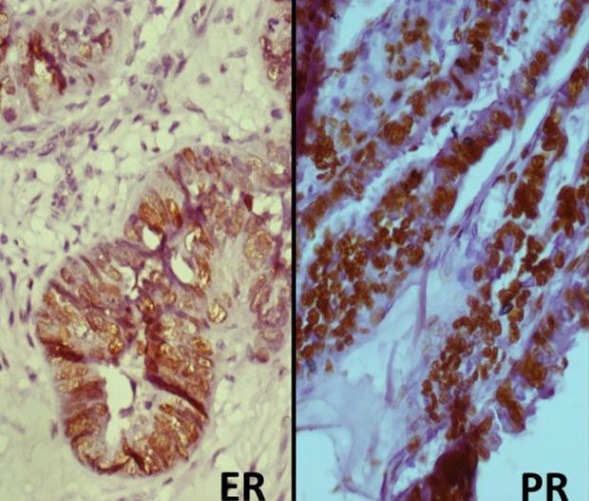
Photomicrographs showing strong nuclear immunostaining with ER (left, X400) and strong nuclear immunostaining for PR (right, X400)

**Immunohistochemistry**: Immunohistochemical stains were applied to the sections according to the earlier described method [21]. Five-μm sections were obtained from each of the tissues and stained with ER (DAKO USA, clone 1D5; 1:25) and PR (DAKO USA, clone PgR636; 1:50) antibodies. The five micrometre tissue sections attached on silanized slides were baked, deparaffinised in xylene, rehydrated in graded ethanol (100% and 95%), rinsed in water and covered with Tris buffers. These slides were then incubated for 20 minutes with primary monoclonal antibodies against ER (DAKO, clone 1D5; 1:25) and PR (DAKO, clone PgR636; 1:50) followed by incubation with biotin-labelled secondary antibodies, a polyclonal goat anti-mouse antibody for both ER and PR (DAKO USA, REF: K0675, LOT: 10081219). The streptavidin-peroxidase complex was visualized using di-aminobenzidine as a chromogenic substrate. Breast tissue was used as a positive control. Slides were reviewed by two of the authors (MAA and AOO) and discordant scores were resolved through a consensus scoring. The agreement among the observers was 95%. Only nuclear staining of the tumour cells was considered a positive expression for ER and PR (Figure 1). Grading of nuclear ER and PR staining was performed using an immunoreactive H-scoring system obtained by the product of intensity of immunostaining (none = 0 (negative); 1-25% = 1+ (weak); 26-50%=2+ (moderate); > 50% = 3+(strong)). The ER and PR positivity was defined as ≥ 1% tumour cell nuclei (i.e. encompassing weak, moderate and strong nuclear staining) [22]. The data obtained were subjected to statistical analysis using Statistical Package for Social Sciences (SPSS) version 20. Statistical analysis was used to evaluate statistical associations between expression of ER and PR, clinicopathological parameters i.e. age, stage, grade, and histological subtypes. Continuous variables were compared using the student's T test and categorical variables were compared using the chi-square test. Statistical significance was defined as p < 0.05. This study was reviewed and approved by the Human Research Review Committee of the Joint University of Ibadan/University College Hospital.

## Results

The age range of patients from which the surgical specimens were obtained was from 16 years to 82 years with a mean age of 52.2 ± 12.6 years. The peak age of occurrence of epithelial ovarian cancers was in the fifth decade of life. There was only one case of epithelial ovarian cancer seen in the first two decades of life. Serous carcinomas was the most common histological subtype and was seen in 63 (70.0%) cases, followed by mucinous carcinoma, which accounted for 24 (26.7%)cases, There were 2 (2.2%) endometrioid carcinomas and only 1(1.1%) case of malignant Brenner tumour (Table 1). The poorly differentiated (grade 3) serous carcinomas were more commonly seen while the reverse is the case in mucinous carcinomas with grade 1 tumours being more common (Table 2). ER and PR are more expressed (35% and 53% respectively) in grade 3 serous carcinoma compared with mucinous carcinoma which has highest PR expression (18%) in grade 2 tumours (Table 2). A slight majority of Forty-seven (52.2%) cases of epithelial ovarian cancer were at advanced FIGO stage (III/IV) and while forty-three (47.8%) cases were at early FIGO stage (I/II). Nineteen (21.1%) cases were well-differentiated (grade 1), 34 (37.8%) were moderately differentiated (grade 2) while 37 cases (41.1%) were poorly differentiated (grade 3). Eight (12.7%) out of 63 cases of serous carcinoma were grade 1 (well differentiated serous carcinomas) tumours. The expression rate of ER and PR in the 90 ovarian carcinomas was 24.4% (22 cases) and 33.3% (30 cases) respectively. There was a positive correlation between PR and ER expression (p = 0.001). A significant positive correlation was also found between histological grade and PR expression (p = 0.027). No significant association was found between grade and ER expression (p = 0.125). Equal number, 11 (50%), of ER expression rate was found both in early stage (I/II) and in advanced stage (III/IV) whereas more PR expression rate 19 (63.3%) were found in advanced stage (III/IV). A higher proportion of ER negative are in stage III/IV. No significant association was found between FIGO stage and ER and PR expression (p = 0.728 and 0.258 respectively). The majority of cases of serous carcinoma and mucinous carcinoma were negative for ER and PR (69.8% and 58.7% respectively). On the average, significantly fewer carcinomas were negative for ER. No association was found between PR expression and histological subtypes (p = 0.650) (Table 3).

**Table 1:** Histological subtypes

Histological subtypes	Number	%
Serous carcinoma	63	70.0
Mucinous carcinoma	24	26.7
Endometrioid carcinoma	2	2.2
Malignant Brenner tumour	1	1.1

**Table 2:** Hormone receptor expression of different histotypes of epithelial ovarian cancer with histological grades

Histological grades						
	Grade 1		Grade 2		Grade 3	
**Serous carcinoma**						
	Positive	Negative	Positive	Negative	Positive	Negative
**ER**	2	6	5	16	12	22
**PR**	2	6	6	15	18	16
**Mucinous carcinoma**						
	Positive	Negative	Positive	Negative	Positive	Negative
**ER**	1	9	0	11	1	2
**PR**	1	9	2	9	0	3
**Endometrioid carcinoma**						
	Positive	Negative	Positive	Negative	Positive	Negative
**ER**	0	0	0	0	0	0
**PR**	0	0	1	1	0	0
**Malignant Brenner tumour**						
	Positive	Negative	Positive	Negative	Positive	Negative
ER	1	0	0	0	0	0
PR	0	0	0	0	0	0

**Table 3:** Relationship between histopathological subtypes and ER and PR expressions

Histological subtypes	Serous carcinoma	Mucinous carcinoma	Endometrioid carcinoma	Malignant Brenner tumour	Total	P-value
**ER**						
Negative	44 (69.8%)	22 (91.7%)	2 (100.0%)	0	68	
Positive	19 (30.2%)	2 (8.3%)	0	1 (100.0%)	22	0.042
**PR**						
Negative	37 (58.7%)	21(87.5%)	1 (50.0%)	1 (100.0%)	60	
Positive	26 (41.3%)	3 (22.5%)	1 (50.0%)	0	30	0.650
**Total**	63 (100%)	24 (100%)	2 (100%)	1 (100%)	90 (100%)	

## Discussion

The expression rates of ER and PR in this study is similar to what was reported by Ayadi et al from Tunisia where ER and PR were expressed in 35.1% and 33.3% of EOC respectively [17]. In the literature, a wide range of steroid receptor expression in EOC has been reported, namely 32-77% for ER and 15-69% for PR [5, 17, 18, 23–35 ]. The high variability in the percentage of receptor positivity may be related to the different assay methods employed in the different studies. Generally, higher figures for steroid receptor expression were obtained in earlier studies using biochemical techniques or flow cytometry, as compared to more recent studies using Immunohistochemistry [17, 23–28, 30, 31, 33]. Kommoss et al from Germany recorded ER and PR positivity of 38% and 31% respectively while Ayadi et al from Tunisia recorded ER and PR positivities of 35.1% and 33.3% respectively which compares favourably with the findings of the present study [17, 32]. There was a significant association between ER and the histological subtypes (p = 0.042). A higher proportion of serous (30%) than mucinous (8%) carcinomas were observed to be ER positive. There is a stronger association with serous subtype compared with the mucinous types. This agrees with what was found by Ayadi et al from Tunisia and Arias-Pulido et al from Mexico [17, 36]. There was a significant ER negative expression in mucinous carcinoma in this study which corroborate with what has been reported in the literature [17, 18, 36, 37 ]. However majority of cases (69.8%) of serous carcinoma were ER negative. This finding contrasts with the findings of Arias-Pulido et al who reported that majority of cases of serous EOC were ER-positive (66.7%) [36]. In the current study, although 59.1% of ER positive epithelial ovarian cancer were poorly differentiated (grade 3), and 18.2% were well differentiated (grade 1), no significant association was found between grade and ER expression. The findings in this study contrast with what was found in other studies where ER was found to be more expressed in grade 1 tumours than grade 3 tumours [17, 38].

There was no significant association between ER expression and FIGO stage in our study similar to what was reported by Kauppila et al [37]. This however contrasts with the report of Burges et al where a significant expression of ER was observed in advanced FIGO stage [35]. Kauppila et al also reported an association between ER expression, age and grade similar to what was reported in this study [37]. There was positive correlation between ER and PR expression. This observation compares with what was found by Arias-Pulido H et al from Mexico and Ayadi et al from Tunisia, who also found positive correlation between ER and PR expression [17, 36]. In this study, a significantly greater proportion of both serous/endometrioid carcinomas (41.3% and 50% respectively) than mucinous carcinomas (22.5%) were PR positive. This finding is similar to what was found by Arias-Pulido et al [36] where PR positive expression was greater in serous (54.9%) and endometrioid (71.4%) than in mucinous carcinomas (20%). Other studies have confirmed that the majority of mucinous ovarian carcinomas are PR negative [17, 23, 24, 29, 32, 33]. In the current study, a significant positive correlation was found between tumour grade and PR expression, with 60% of PR positive epithelial ovarian cancers being grade 3 (poorly differentiated) tumours, and 10% being grade 1 (well differentiated) tumours. This finding is in agreement with some studies [29, 38, 39] but however, contrasts with what was found in other studies where PR was found to be more expressed in grade 1 tumours than grade 3 tumours [17, 24, 37]. There was no significant association between PR expression and age. This finding is in contrast what was found by Spona et al and Scambia et al who demonstrated a higher incidence of PR positive EOC in patients older than 60 years of age [26, 39]. There was no association between PR expression and FIGO stage. These findings are in agreement with some reports in the literature [29, 39] but however contrast with Ayadi et al who observed that PR expression is correlated with early stage of EOC [17].

## Conclusion

This study has shown significant correlations between PR expression and increasing histological grade and between ER expression and serous histological subtype as compared with mucinous variant. These findings suggest that hormone receptors status may be of potential benefit in the management of patients with EOC. It is therefore recommended that future studies employing larger sample size and stratified by histological subtype and biomarker status are required to establish whether or not ER or PR status of EOC could predict response to endocrine therapy.

### What is known about this topic

- Majority of cases of serous carcinoma were positive for estrogen receptor;
- Estrogen receptor and progesterone receptor have been found to be more expressed in grade 1 tumours than grade 3 tumours.

### What this study adds

- Majority of cases of serous carcinoma were negative for estrogen receptor;
- Progesterone receptor was found to be more expressed in grade 3 tumours than grade 1 tumours whereas there was no significant association between the grade and estrogen expression.

## Competing interests

The authors declare no competing interest.
